# The gut microbiota composition in patients with right- and left-sided colorectal cancer and after curative colectomy, as analyzed by 16S rRNA gene amplicon sequencing

**DOI:** 10.1186/s12876-022-02382-y

**Published:** 2022-06-25

**Authors:** Daisuke Suga, Hiroki Mizutani, Shunsuke Fukui, Mayu Kobayashi, Yasuaki Shimada, Yuuichi Nakazawa, Yuuki Nishiura, Yuuya Kawasaki, Isao Moritani, Yutaka Yamanaka, Hidekazu Inoue, Eiki Ojima, Yasuhiko Mohri, Hayato Nakagawa, Kaoru Dohi, Kei Takaba, Hideo Wada, Katsuya Shiraki

**Affiliations:** 1 Department of Gastroenterology, Mie General Medical Center, Yokkaichi, 510-8561 Japan; 2 Department of Surgery, Mie General Medical Center, Yokkaichi, 510-8561 Japan; 3 Department of Gastroenterology, Mie University Graduate School of Medicine, Tsu, 514-8507 Japan; 4 Department of Cardiology and Nephrology, Mie University Graduate School of Medicine, Tsu, 514-8507 Japan; 5 Department of Research Center, Mie General Medical Center, Yokkaichi, 510-8561 Japan; 6 Department of Clinical Medicine, Mie Graduate School of Medicine, Mie General Medical Center, Yokkaichi, 510-8561 Japan

**Keywords:** Colon cancer, Microbiota, T-RFLP, 16S rRNA gene amplicon sequencing

## Abstract

**Background:**

Gut pathological microbial imbalance or dysbiosis is closely associated with colorectal cancer. Although there are observable differences in molecular and clinical characteristics between patients with right- and left-sided colon cancer, differences in their gut microbiomes have not been thoroughly investigated. Furthermore, subsequent changes in microbiota status after partial colectomy remain unknown. We examined the human gut microbiota composition to determine its relationship with colon cancer and partial colon resection according to location.

**Methods:**

Stool samples from forty-one subjects (10 in the control group, 10 in the right-sided colon cancer [RCC] group, 6 in the sigmoid colon cancer [SCC] group, 9 in the right colon resection [RCR] group and 6 in the sigmoid colon resection [SCR] group) were collected, and DNA was extracted. After terminal restriction fragment length polymorphism (T-RFLP) analysis, the samples were subjected to 16S rRNA gene amplicon sequencing, and the metabolic function of the microbiota was predicted using PICRUSt2.

**Results:**

T-RFLP analysis showed a reduced ratio of *clostridial cluster XIVa* in the SCC patients and *clostridial cluster IX* in the RCC patients, although these changes were not evident in the RCR or SCR patients. 16S rRNA gene amplicon sequencing demonstrated that the diversity of the gut microbiota in the RCC group was higher than that in the control group, and the diversity in the SCR group was significantly higher than that in the RCR group. Principal coordinate analysis (PCoA) revealed significant differences according to the group. Analyses of the microbiota revealed that *Firmicutes* was significantly dominant in the RCC group and that the SCC group had a higher abundance of *Verrucomicrobia*. At the genus level, linear discriminant analysis effect size (LEfSe) revealed several bacteria, such as *Ruminococcaceae*, *Streptococcaceae*, *Clostridiaceae*, *Gemellaceae*, and *Desulfovibrio*, in the RCC group and several oral microbiomes in the SCC group. Metabolic function prediction revealed that cholesterol transport- and metabolism-related enzymes were specifically upregulated in the RCC group and that cobalamin metabolism-related enzymes were downregulated in the SCC group.

**Conclusion:**

Gut microbial properties differ between RCC and SCC patients and between right hemicolectomy and sigmoidectomy patients and may contribute to clinical manifestations.

## Introduction

There is emerging evidence that gut pathological microbial imbalance or dysbiosis occurs in patients with colorectal cancer [1–5]. Changes in the intestinal microbiome can promote chronic inflammatory conditions and the production of carcinogenic molecules, subsequently increasing the risk of colorectal cancer. Specific alterations of the microbiome are observed during different stages of colorectal cancer [6–8]. These bacteria include *Fusobacterium*, *Peptostreptococcus*, *Porphyromonas*, *Prevotella*, *Parvimonas*, *Bacteroides*, *Gemella* and the oral microbiome. Their metabolism or immune modulation may directly or indirectly affect the colonic mucosal cells, causing colonic carcinogenesis [2–5]. However, how gut microbial dysbiosis may be involved in cancer pathogenesis remains undescribed.

In general, colorectal cancer pathogenesis depends on the location of the tumor. The proximal colon (right side) and distal colon (left side) exhibit different molecular characteristics and histologies. Right-sided tumors generally demonstrate flat histology associated with mutations in the DNA mismatch repair pathway. In contrast, left-sided tumors demonstrate epolypoid-like morphology with chromosomal instability-related mutations [9–11]. These findings raise new insights that the colorectal cancer-related gut microbiome may differ among tumor locations. However, few studies have analyzed microbiome changes between right- and left-sided colon cancers [12, 13].

However, a nationwide study suggested that the risk of diabetes mellitus (DM) is high in colectomy patients, especially in patients who received resection of the left part of the colon and the sigmoid colon, whereas resection of the rectum was not associated with a risk of DM [14]. These results suggest that the left colon may play a role in the regulation of glucose homeostasis. In addition, patients with left hemicolectomy were at higher risk of cerebrovascular disease [15]. These results indicate that different colectomy procedures may influence metabolic diseases after colectomy.

It is known that dysbiosis and alterations in gut microbial composition are linked to the development of metabolic diseases, including DM [16, 17]. In previous reports, patients with DM had enriched *Clostridium clostridioforme* and *Lactobacillus* species but low *Roseburia*, a major butyrate producer. The ratio of *Bacteroidetes* to *Firmicutes* demonstrated a significantly positive correlation with reduced glucose tolerance, which suggests that the gut microbiome may play an important role in the development of DM [18–20].

From this evidence, we suppose that the change in gut microbiota after colectomy may be involved in the regulation of multiple metabolic, signaling, and inflammatory pathways that are related to general physiological conditions. However, the gut microbiota after different sites of colectomy has not been well elucidated.

Therefore, we first investigated the gut microbiota in patients with right-sided colon cancer (RCC) and sigmoid colon cancer (SCC) to elucidate the different microbiota that may be responsible for colon carcinogenesis between different sites. We also analyzed the gut microbiota in patients with right colon resection (RCR) and with sigmoid colon resection (SCR) to reveal long-term changes in the microbiota after different colon resections. Finally, we aimed to provide new insights into the microbiota status of colon cancer and subsequent colectomy at different sites.

## Methods

### Human subjects

Subjects who were under 77 years of age and had undergone colonoscopy at the Mie Prefectural General Medical Center, Yokkaichi, Japan, between 2017 and 2020 were enrolled in the study. To evaluate differences in gut microbiota via terminal restriction fragment length polymorphism (T-RFLP) analysis, the subjects were classified into five groups: (i) control subjects who had no history of bowel disease; (ii) patients who had recently been diagnosed with colon cancer from the cecum to the transverse colon and who were awaiting surgery (the RCC group); (iii) patients who had recently been diagnosed with sigmoid colon cancer and were awaiting sigmoid colectomy (the SCC group); (iv) patients with a diagnosis of colorectal cancer undergoing right colon resection (the RCR group); and (v) patients with a diagnosis of sigmoid colon cancer undergoing sigmoid colon resection (the SCR group). We excluded all participants with a current use of antibiotics, history of current chronic bowel or liver disease, history of chemotherapy, and regular use of immunomodulators or probiotics. Patient assignments are shown in Table 1. Stool samples were collected from each participant, and fecal samples were stored at 4 °C until analysis.

**Table 1:** Demographic and clinical characteristics of the study group

	Control (n = 10)	RCC (n = 10)	SCC (n = 6)	RCR (n = 9)	SCR (n = 6)
Age; years	56.1 ± 5.7	70.3 ± 11*	63.3 ± 11	68.6 ± 9.2*	61.0 ± 2.1
Gender, male; n (%)	6 (60)	4 (40)	3 (50)	5 (55.6)	3 (50)
BMI; kg/m^2^	21.4 ± 4.2	23.1 ± 5.7	25.2 ± 4.4*	24.5 ± 5.3*	22.0 ± 2.1
Smoking, yes; n (%)	5 (50)	1 (10)	2 (33.3)	5 (55.6)	2 (33.3)
Alcohol, yes; n (%)	8 (80)	3 (30)	2 (33.3)	6 (66.7)	3 (50)
Diabetes, yes; n (%)	0 (0)	1 (10)	0 (0)	2 (22.2)	1 (16.7)
Hypertension, yes; n (%)	3 (30)	6 (60)	4 (66.7)	2 (22.2)	3 (50)
Total cholesterol; mg/dl	195.7 ± 25.5	188 ± 0.7	186 ± 8.5	205.4 ± 19.8	194.8 ± 7.1
Triglyceride; mg/dl	86.8 ± 33.2	97.6 ± 75	127.3 ± 14.1	94.8 (5/9)	99 (2/6)
HDL cholesterol; mg/dl	66.5 ± 20.5	87.6 ± 14.1	50.6 ± 12	61.4 (5/9)	83.5 (2/6)
LDL cholesterol; mg/dl	113.2 ± 7.8	103.9 ± 6.4	111.2 ± 17.7	111.2 (5/9)	93.5 (2/6)

Means ± SDs; *P*-values are based on Kruskal–Wallis test for continuous variables and Fisher’s exact test for categorical variables; BMI, body mass index; HDL, high-density lipoprotein; LDL, low-density lipoprotein. *P < 0.05 vs. the control

### DNA extraction

DNA extraction was performed using a previously described method [7]. In brief, an aliquot of the suspension of fecal samples was homogenized with zirconia beads in a 2.0-ml screw cap tube by a FastPrep 24 Instrument (MP Biomedicals, Santa Ana, CA, USA). We extracted DNA from each sample using an automatic nucleic acid extractor (Precision System Science, Chiba, Japan). We used MagDEA 200 (GC; Precision System Science) as the reagent for automated nucleic acid extraction.

### T-RFLP

T-RFLP analysis of the microbial community structure in feces was performed by TechnoSuruga Laboratory Co., Ltd. (Shizuoka, Japan). We performed amplification of the bacterial 16S rRNA gene (16S rDNA), restriction enzyme digestion, size fractionation of fluorescently labeled terminal restriction fragments (T-RFs) and T-RFLP data analysis by a previously described method by Nagashima et al. [21, 22]. Briefly, the 5′ HEX-labeled 516 f and 1516 r primers were used for amplification of the bacterial 16S rRNA. Then, the PCR products were digested with 10 U of *Bsl*I (New England BioLabs, Ipswich, MA, USA). The resultant DNA fragments, namely, fluorescently labeled T-RFs, were analyzed by an ABI PRISM 3130xl genetic analyzer, and their length and peak area were determined using the genotype software GeneMapper (Applied Biosystems). T-RFs were divided into 29 operational taxonomic units (OTUs). The OTUs were quantified as the percentage of individual OTUs per total OTU area, which was expressed as the percentage of the area under the curve (%AUC). The bacteria were predicted for each classification unit, and the corresponding OTUs were identified according to reference Human Fecal Microbiota T-RFLP profiling (https://www.tecsrg.co.jp/t-rflp/t_rflp_hito_OTU.html).

To evaluate differences in gut microbiota composition at the species level, samples from 10 control subjects and 31 patients with carcinoma were selected for 16S rRNA gene amplicon sequencing; StatView-J 5.0 was used to match control and patient samples based on age, sex, BMI, smoking, alcohol, diabetes, hypertension, total cholesterol, triglyceride, HDL cholesterol, and LDL cholesterol.

### Illumina library generation

Next-generation sequencing (NGS) analysis of the microbial community structure in the feces was performed using a MiSeq (Illumina, San Diego, CA, USA), as previously described by Takahashi et al. [23]. The V3-V4 regions of bacterial and archaeal 16S rRNA were amplified using the Pro341F/Pro805R primers and the dual-index method [23]. Barcoded amplicons were paired-end sequenced on a 2 × 284-bp cycler using the MiSeq system together with MiSeq Reagent Kit version 3 (600 cycles).

### Quality filtering and amplicon sequencing analysis

Paired-end sequencing reads were merged using the fastq-join program with default settings. Only combined reads with a quality value score of ≥ 20 for more than 99% of the sequence were extracted using FASTX-Toolkit. The chimeric sequences were deleted with USEARCH ver. 6.1 [24]. Identification from sequence analyses of sequence reads was performed manually using the Ribosomal Database Project (RDP) Multiclassifier tool ver. 2.11, which is available from the RDP website (http://rdp.cme.msu.edu/classifier/). Bacterial and archaeal species identification from sequences was performed using Metagenome@KIN 2.2.1 analysis software (World Fusion, Japan) and the TechnoSuruga Lab Microbial Identification database DB-BA ver. 13.0 (TechnoSuruga Laboratory, Japan) with homology ≥ 97% [25]. Principal coordinate analysis (PCoA) was performed using Metagenome@KIN software (World Fusion Co., Ltd., Tokyo, Japan) based on data from bacterial genera with a 97% similarity cutoff with the Apollon DB-BA database ver. 13.0 (TechnoSuruga Laboratory).

### Alpha and beta diversity analysis

The joined amplicon sequence reads were processed through QIIME 2 ver. 2020.6. Quality filtering and chimeric sequences were filtered with the default option, and representative sequences were created using the DADA2 denoise-single plugin ver. 2017.6.0. The taxonomy of representative sequences was assigned using the Greengenes database ver. 13.8 by training a naive Bayes classifier using the q2-feature classifier plugin. The sampling depth for alpha and beta diversity was 28,216, which was the minimum number of read counts among samples.

Alpha diversity indices (Chao1, Shannon and Simpson) were calculated using the alpha rarefaction plugin. The statistical significance of the Chao1, Shannon and Simpson indices among the groups was assessed by the Kruskal–Wallis test using the alpha-group-significance plugin.

Beta diversity was analyzed using weighted UniFrac, unweighted UniFrac and Bray–Curtis distances using a core-metrics-phylogenetic plugin. The Emperor tool was used to visualize the PCoA plots. The statistical significance of the similarity of bacterial communities among groups was assessed with the ANOSIM test using the beta-group-significance plugin.

### Predictive functionality analysis

Predictive functionality analysis of bacterial 16S rDNA communities was performed using PICRUSt ver. 2.3.0-b. The analyzed representative sequences by QIIME 2 and the reference sequence of the Integrated Microbial Genomes database (IMG) were aligned using HMMER ver. 3.3. Phylogenetic placement analyses were applied using EPA-NG ver. 0.3.3 and GAPPA ver. 0.6.0. 16S rRNA gene copies were normalized using the caster package of R software. The gene families were predicted based on the Clusters of Orthologous Genes (COG) and Enzyme Classification (EC) databases. Biological pathways were reconstructed based on predicted gene families using MinPath ver. 1.4 [26].

### Linear discriminant analysis (LDA)

To determine potential bacteria that differ in abundance between the groups, a linear discriminant analysis effect size (LEfSe) analysis in multilevel species was used. LEfSe can identify taxa with significantly normalized relative abundances and performs a linear discriminant analysis (LDA) to determine the effect size of each taxon through the website http://huttenhower.sph.harvard.edu/galaxy [27]. Taxa with an effect size greater than 2.0 (with *P* < 0.05) were considered significant.

Metabolic function prediction of the microbiota by PICRUSt2.

The mean (standard deviation [SD]) and median (interquartile range [IQR]) were used to describe normally and nonnormally distributed data, respectively. Numbers and percentages were calculated for categorical variables. The data were analyzed using the Kruskal–Wallis test or the Mann–Whitney test (two-sided) for continuous variables and Fisher’s exact test for categorical variables using StatView-J 5.0. *P* values < 0.05 were considered significant.

## Results

### Clinical patient characteristics

The demographic and clinical characteristics of the subjects are shown in Table 1. Ten healthy controls and 31 patients with cancer (breakdown: RCC, 10 patients; SCC, 6 patients; RCR, 9 patients; and SCR, 6 patients) were enrolled. The average postoperative observation period was 37 months (minimum 13 months, maximum 90 months). Blood tests showed no significant differences in total cholesterol, triglycerides, HDLs, or LDLs in any group, including the control group. The mean age and BMI of the healthy subjects were lower than those of the cancer patients. Two diabetic patients were found in the RCR group and one in the SCR group.

### Differences in microbiota between control and cancer patients by T-RFLP analysis

The results of T-RFLP are shown in Table 2. *Lactobacillales* were more abundant in all the groups than in the control group. *Bifidobacterium* tended to increase in the RCR and SCR groups, while *Prevotella* tended to decrease in the RCR and SCR groups. In the *Clostridium cluster*, a decrease in *Clostridium cluster XIVa* increased in the SCC group (*P* < 0.05). In addition, *Clostridium cluster IX* decreased in the RCC group (*P* < 0.05). The postoperative RCR and SCR groups of both *Clostridium cluster IX* and *XIVa* were not different from those of the control group.

**Table 2:** Differences in bacterial microbiota according to T-RFLP analysis

	Control (n = 10)	RCC (n = 10)	SCC (n = 6)	RCR (n = 9)	SCR (n = 6)
Bifidobacterium	6.54 ± 6.75	5.06 ± 7.14	8.18 ± 10.83	9.23 ± 7.96	15.7 ± 12.15
Lactobacillales	0.94 ± 0.88	9.76 ± 8.56*	6.70 ± 9.02	8.78 ± 10.85*	3.72 ± 2.88*
Bacteroides	49.7 ± 10.6	37.5 ± 18.9	52.5 ± 23.1	50.1 ± 11.2	47.4 ± 17.3
Prevotella	5.19 ± 9.92	7.93 ± 14.1	7.98 ± 14.9	1.19 ± 2.87	0.12 ± 0.29
Clostridium IV	5.87 ± 6.85	12.1 ± 9.3**	3.2 ± 3.17	2.97 ± 2.49**	4.7 ± 2.05
Clostridium XIVa	15.7 ± 4.14	15.7 ± 6.97	11.4 ± 3.37*,**	15.6 ± 7.5	17.5 ± 4.14**
Clostridium IX	8.54 ± 8.1	1.33 ± 2.13*	2.6 ± 3.19	3.96 ± 8.3	2.25 ± 2.81
Clostridium XI	0.19 ± 0.41	0.73 ± 1.3	0.23 ± 0.48	0.18 ± 0.53	1.25 ± 1.49*
Clostridium XVIII	1.98 ± 1.8	2.27 ± 2.76	1.35 ± 1.02	1.22 ± 0.96	1.33 ± 1.44
Other	5.42 ± 2.74	7.57 ± 3.56	5.88 ± 4.75	6.79 ± 4.96	6.0 ± 3.15

*P*-values are based on the Kruskal–Wallis test; the data are expressed as the means ± SDs; T-RFLP, terminal restriction fragment length polymorphism. *P < 0.05 vs. the control; **Significant difference between the preoperative and postoperative groups (*P* < 0.05)

### Differences in species richness and diversity between control and cancer subjects

**Fig. 1:**
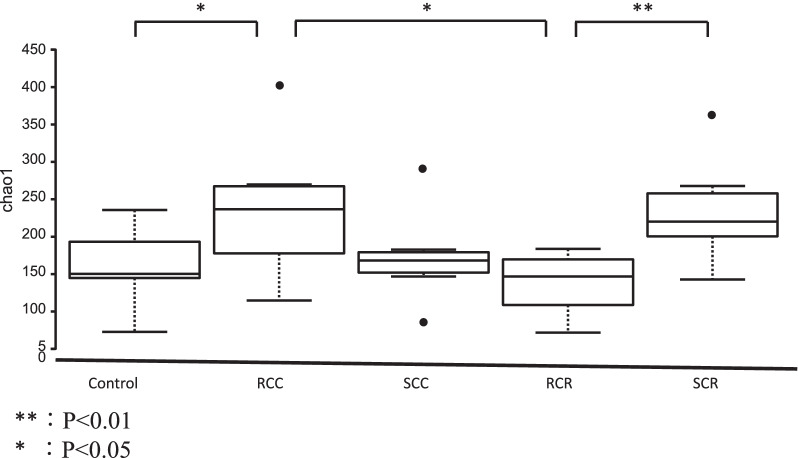
Richness and diversity analysis of 16S rRNA gene amplicon sequences obtained from fecal samples. The Chao1 index was used to evaluate microbial richness and diversity in fecal samples between patients in the healthy control and cancer groups (*P < 0.05, **P < 0.01)

Because characteristic changes in the gut microbiota were observed in each group, 16S rRNA gene amplicon sequencing was performed to analyze the microbiota in more detail. First, we examined diversity. The Chao1 index was applied to assess the influence and diversity of the microbiota in the feces between the control group and cancer patients. Figure 1 shows the alpha diversity between each group, including the control group. Chao1 showed a predominant increase in diversity in the RCC group but not in the SCC group; the RCR group was less diverse than the RCC group (*P* < 0.05). In addition, the SCR group was more diverse than the RCR group (*P* < 0.01). Similar results were obtained with Shannon and Simpson analyses.

**Fig. 2:**
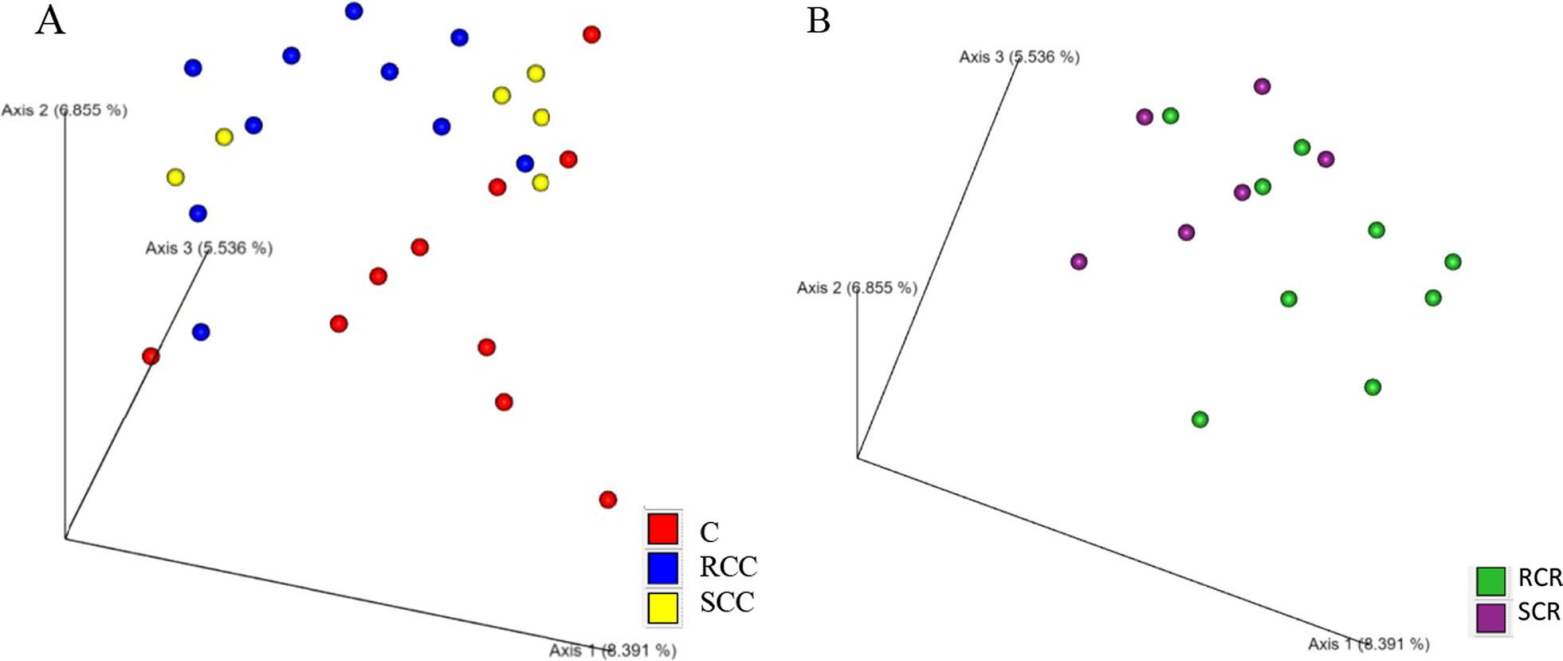
Beta diversity was assessed by the Bray–Curtis test. In (**A**), the red dots indicate the control group, the blue dots indicate the RCC group, and the yellow dots indicate the SCC group. In (**B**), the green dots indicate the RCR group, and the purple dots indicate the SCR group. The beta diversity represents the degree of difference in diversity between two samples. The Bray–Curtis distance was computed via the 16S rRNA gene amplicon sequence data. (*P* values: *P* < 0.01 for the control group and RCC group; *P* < 0.05 for the control group and SCR group; and *P* < 0.01 for the RCC group and RCR group. *P* = 0.095 for the RCR group and SCR group)

PCoA was then performed to visually assess diversity. Figure 2 shows the beta diversity between each cancer group, including the control group. In Bray–Curtis ANOVA, there was a significant and clear cluster difference between the control group and the RCC group (*P* < 0.01), the control group and the SCR group (*P* < 0.05), and the RCC group and the RCR group (*P* < 0.01). There was no significant difference between the RCR and SCR groups, but the composition tended to be different (P = 0.095). Similar results were observed with weighted UniFrac and unweighted UniFrac with other analysis methods. In the unweighted UniFrac analysis, the P value for the comparison of the RCR and SCR groups was 0.001.

### 16S rRNA gene amplicon sequencing in each systematic classification

**Fig. 3:**
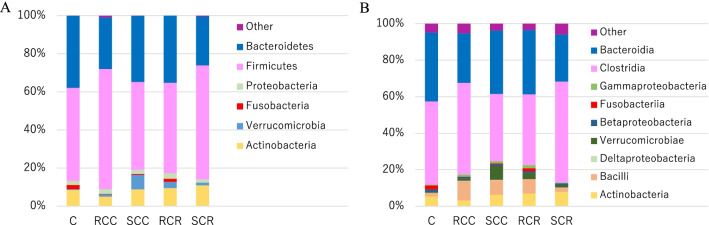
Phylum (**A**)- and class (**B**)-level classifications of bacteria identified in individual fecal samples of the control group and each cancer group. Each bar represents the percent contribution of phylum- and class-level profiles. The phylum and class represented by the different colors are shown below the figure

Figure 3 shows the relative proportions of gut microbiota at the phylum and class classification levels in each group. As shown in Fig. 3A, *Firmicutes* was more abundant in the RCC group than in the other groups, including the control group, and the SCC group had more *Verrucomicrobia. Bacteroidetes* decreased in the SCR group compared to the control group. As shown in Fig. 3B, *Bacilli* was more abundant in the RCC group, and *Verrucomicrobia* was more abundant in the SCC group than in all the other groups, including the control group. *Clostridia* increased in the postoperative SCR group compared to the SCC group. *Fusobacteriia* was not significantly different between groups, including the control group, even at the class level.

### Comparison of bacterial classifications between control and cancer patients

**Fig. 4:**
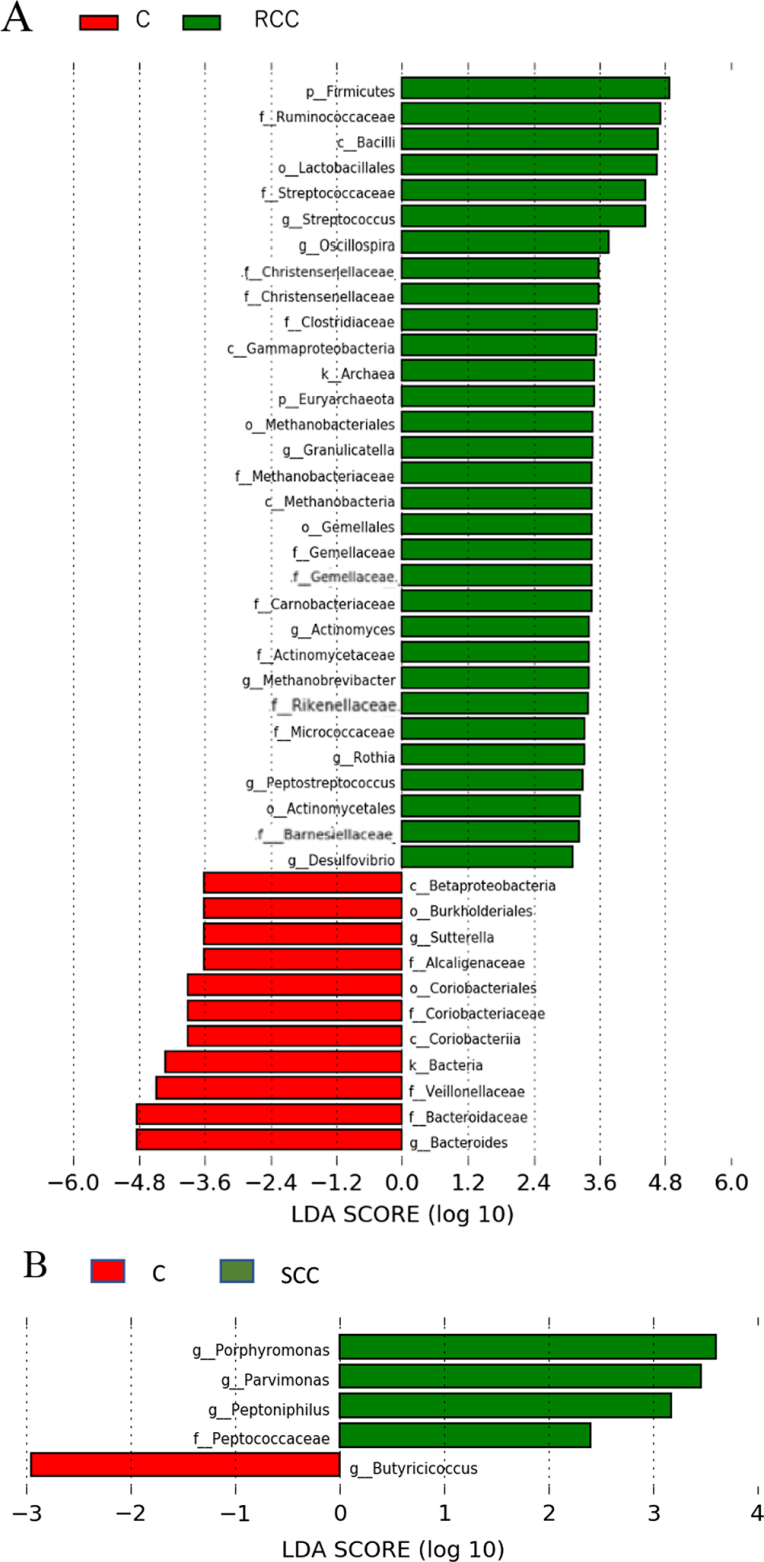

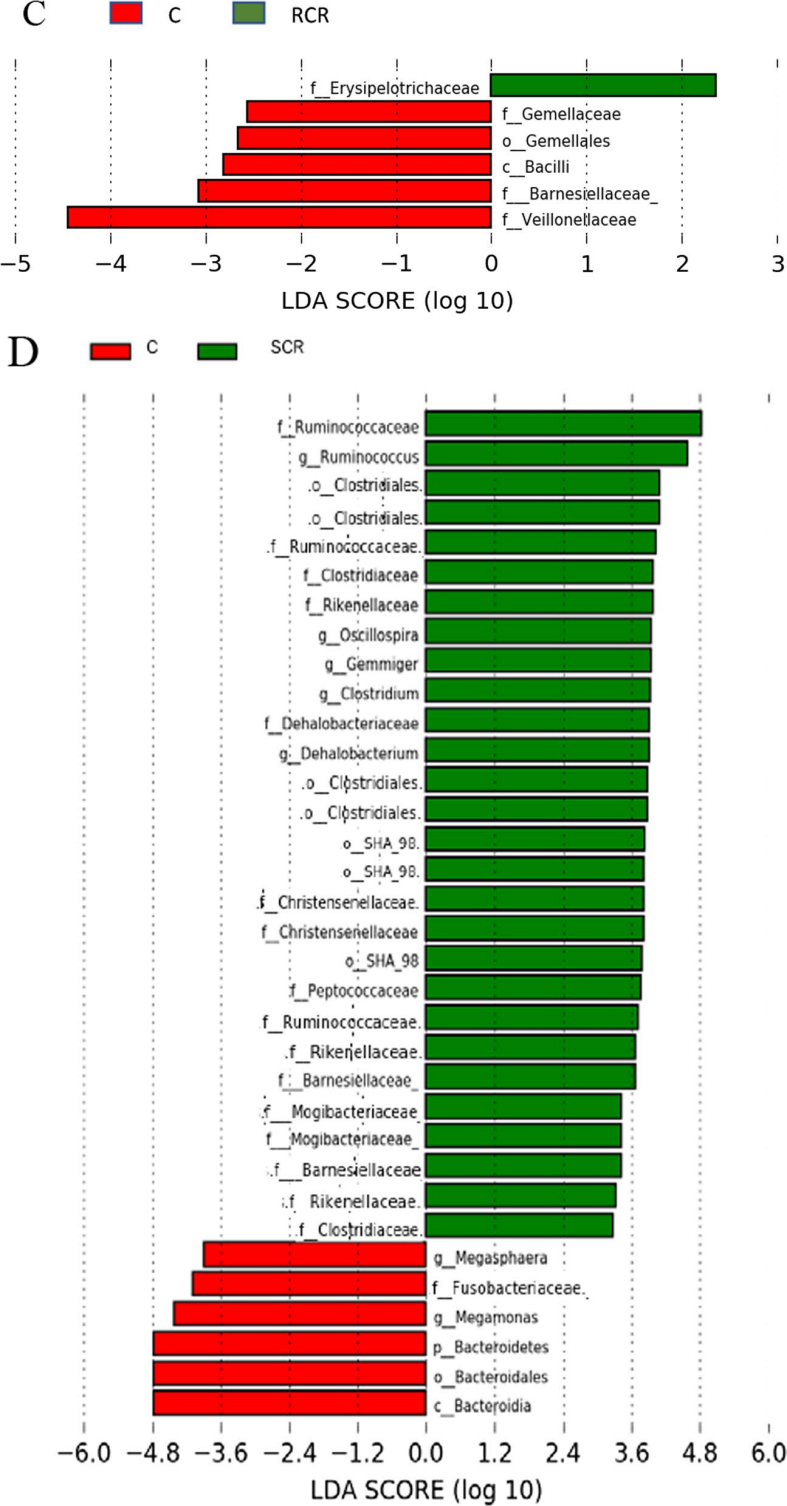
LEfSe comparing the control and bacterial classifications of tumor-related microorganisms. The histograms of LDA scores for differentially abundant bacterial groups are shown in red for the control group and green for the cancer patient group. The control group and RCC group analyses are designated (**A**); the control group and SCC group analyses are designated (**B**); the control group and RCR group analyses are designated (**C**); and the control group and SCR group analyses are designated (**D**). Each analysis was performed at the genus level, but when the genera were not clear, the next level of hierarchy was used

Next, a linear discriminant analysis (LDA) effect size (LEfSe) approach was performed to clarify the characteristic genus-level bacteria in each group, as shown in Fig. 4. LEfSe can determine the taxonomic units most likely to explain differences between classes by coupling standard tests for statistical significance with additional tests encoding biological consistency and effect relevance (LDA score > 2.0, P < 0.05). An increase in the abundance of thirty-one genera was detected in the RCC group. Among them, *Ruminococcaceae*, *Gemella*, *Streptococcaceae*, *Peptococcaceae,* and *Desulfovibrio* have been suggested to be associated with carcinogenesis. On the other hand, eleven genera whose abundance decreased were identified. Only four bacteria were identified in the SCC group. Among them, *Porphyromonas* and *Parvimonas* ranked high among indigenous bacteria in the oral cavity. Only *Butyricicoccus* decreased in abundance. In postoperative patients, the number of bacteria decreased in the RCR group compared to the RCC group. Among the reduced bacteria, *Gemella* abundance decreased. In the SCR group, a large number of bacterial species were identified compared to those in the RCR group. In particular, more bacterial species belonging to the *Ruminococcaceae* family and *Clostridiales* order were identified. *Fusobacteriaceae* was also identified among those bacteria whose abundance was reduced.

### Metabolic function prediction of the microbiota by PICRUSt2

Finally, predictive functionality analysis using PICRUSt2 was performed to clarify the functions of the genes of the microbiota. Tables 3 and 4 show the top 10 genes with the lowest P values among those whose expression increased and decreased in each group. The results revealed many enzymes involved in glucose metabolism, such as glutamyl aminopeptidase and maltose 6`-phosphate phosphatase. Cholesterol-related enzymes were found, especially in the RCC group. However, a decrease in enzymes related to short-chain fatty acids (SCFAs) was observed in the RCC group. The SCC group showed a decrease in vitamin-related enzymes. Moreover, enzymes related to glucose metabolism were found more frequently in the RCR and SCR groups.

**Table 3:** Metabolic function prediction of the microbiota by PICRUSt2 of the RCC and control groups

Variable	*P*-value
*Upregulation (RCC* > *C)*	
Maltose 6′-phosphate phosphatase^) a)^	0.0043
Sulfoquinovose isomerase^a)^	0.0094
D-alanine-poly(phosphoribitol) ligase^a)^	0.0125
Dodecanoyl-[acyl-carrier-protein] hydrolase	0.0127
Phosphoenolpyruvate carboxykinase (GTP)^a)^	0.0135
Mevalonate kinase^b)^	0.0138
DNA-3-methyladenine glycosylase II	0.0141
(2Z,6E)-farnesyl diphosphate synthase^b)^	0.0146
Diphosphomevalonate decarboxylase^b)^	0.0147
Phosphomevalonate kinase^b)^	0.0150
*Downregulation (C* > *RCC)*	
2-Aminoethylphosphonate-pyruvate transaminase	0.0011
Ferredoxin hydrogenase	0.0026
Adenosylmethionine-8-amino-7-oxononanoate transaminase	0.0031
Dethiobiotin synthase	0.0036
UDP-3-O-(3-hydroxymyristoyl)glucosamine N-acyltransferase	0.0040
Kdo(2)-lipid IV(A) lauroyltransferase	0.0041
UDP-3-O-acyl-N-acetylglucosamine deacetylase	0.0042
Fumarate reductase (quinol)^c)^	0.0042
3-Deoxy-8-phosphooctulonate synthase	0.0043
Succinate dehydrogenase (quinone)^c)^	0.0043

Using enzyme classification (EC) numbers, we tabulated the top 10 enzyme-encoding genes with highly significant differences between the RCC and control groups. Each metabolism-related enzyme was classified according to function within the range that could be discriminated as follows. ^a)^ sugar metabolism-related; ^b)^ cholesterol-related; ^c)^ short-chain fatty acid-related

**Table 4:** Metabolic function prediction of the microbiota by PICRUSt2 of the SCC and control groups

Variable	*P*-value
*Upregulation (SCC* > *C)*	
Ribitol-5-phosphate 2-dehydrogenase	0.0005
Lactocepin^c)^	0.0061
Glutamyl aminopeptidase^a)^	0.0067
Licheninase	0.0071
N-acylneuraminate-9-phosphate synthase^a)^	0.0172
Maltose 6′-phosphate phosphatase^a)^	0.0188
Sulfopropanediol 3-dehydrogenase	0.0192
Pyruvate dehydrogenase (acetyl-transferring)^a)^	0.0320
Catechol 2,3-dioxygenase	0.0322
tRNA (uracil(54)-C(5))-methyltransferase	0.0340
*Downregulation (C* > *SCC)*	
Lactaldehyde reductase	0.0051
Selenide, water dikinase	0.0069
Cobyrinate a,c-diamide synthase (glutamine-hydrolyzing)^d)^	0.0081
Hydrogenobyrinic acid a,c-diamide synthase (glutamine-hydrolyzing)	0.0081
Molybdate-transporting ATPase	0.0098
NAD(+) diphosphatase	0.0099
Threonine-phosphate decarboxylase	0.0108
Biotin carboxylase	0.0116
Adenosylcobyric acid synthase (glutamine-hydrolyzing)^d)^	0.0116
Tagatose-bisphosphate aldolase^a)^	0.0120

Using enzyme classification (EC) numbers, we tabulated the top 10 enzyme-encoding genes with highly significant differences between the SCC and control groups. Each metabolism-related enzyme was classified according to function within the range that could be discriminated as follows. ^a)^ sugar metabolism-related; ^c)^ short-chain fatty acid-related; and ^d)^ vitamin-related

## Discussion

In this study, we clearly found that the composition of the gut microbiota differs between right- and left-sided colon cancer patients and after curative colectomy using T-RFLP analysis and 16S rRNA gene amplicon sequencing. These results demonstrated the specific change in gut microbiota in each group, suggesting that gut microbiota are closely associated with the development of colon cancer and physiological conditions after partial colectomy.

T-RFLP analysis showed a reduced ratio of *clostridial cluster XIVa* in the SCC patients and *clostridial cluster IX* in the RCC patients, although these changes were not seen in RCR and SCR patients. *Clostridial cluster XIVa* includes most butyrate producers that belong to the *Firmicutes* phylum in the human colon and *clostridial cluster IX* propionate-producing bacteria [28–30]. SCFAs such as butyrate or propionate are known to have an important role in preserving gut barrier functions and exerting immunomodulatory and anti-inflammatory properties [29, 31]. The antiproliferative, apoptotic and differentiating properties of the various SCFAs are linked to the degree of induced histone hyperacetylation [32]. Fewer SCFAs in feces are observed in patients with IBD or colorectal cancer than in normal patients [33]. Our study indicates that different types of main SCFA-producing bacteria are lower in colon cancer patients and that those of *clostridial cluster IX* are lower in the RCC group and those of *clostridial cluster XIVa* are lower in the SCC group. However, this type of change was not observed in patients after colectomy. Therefore, we speculate that low SCFA-producing bacteria may be related to colon carcinogenesis and are high-risk markers of colon cancer.

We further analyzed the gut microbiota in patients with RCC and SCC and with curative colectomy by 16S rRNA gene amplicon sequencing with QIIME 2, which is a next-generation microbiome bioinformatics platform. We found that the diversity of the gut microbiota in the RCC group was higher than that in the control group, but that in the RCR group was at the same level as that in the control group. In contrast, diversity in the SCR group was significantly higher than that in the RCR group. We also found that gut microbial genus composition using principal component analysis of the log-transformed relative abundances showed a separation between the control group and RCC group and between the RCR group and SCR group. A previous study suggested that patients with colon cancer have a less diverse microbiome than healthy individuals [34]. However, other reports found higher richness in the microbiomes of patients with colon cancer than controls, partly by the expansion of species derived from the oral cavity. A study of microbiota after curative colon surgery suggested that the right hemicolectomy group showed a tendency to decrease in terms of richness and diversity at the genus level [20]. These results are consistent with our current data. The diversity of the gut microbiome is defined as the number and relative abundance distribution of distinct types of microbiomes in the gut. Previous studies have suggested that dysbiosis of the gut microbiome is becoming increasingly recognized for its influence on host immunity and may influence the good response to a variety of cancer therapies, such as chemotherapy, radiotherapy, and immunotherapy [35]. From this point of view, it is very interesting that diversity and richness differed in patients with both pre- and postoperative colorectal cancer according to the tumor location, and high diversity in the SCC group may be associated with favorable outcomes in the SCR group.

Analysis of microbiota at the class level revealed that *Clostridia* and *Bacilli* belonging to *Firmicutes* were significantly dominant in the RCC group compared to the control group. The SCC group had a higher abundance of *Verrucomicrobiae* belonging to *Verrucomicrobia*. These characteristics were not observed in patients in the postcolectomy group. In particular, *Firmicutes* was more abundant and *Verrucomicrobia* was lower in the SCR group than in the RCR group. This tendency was consistent with the findings of a previous long-term study after curative colectomy [20]. These results indicated that there may be specific profiles of the gut bacterial population at the phylum and class taxonomic levels related to colon cancer, and this alteration was different between the locations of cancer. Furthermore, colectomy may also differentially lead to new gut microbiota compositions depending on the removal site.

Next, we performed discriminant analysis by using the LEfSe approach, which was applied to show the key taxa responsible for the difference between several groups and identified several gut microbes mainly at the genus and family levels. Among these bacteria, especially *Ruminococcaceae*, *Streptococcaceae*, *Clostridiaceae*, *Gemellaceae*, and *Desulfovibrio*, which are seen in the RCC group, these species have already been reported to constitute colon cancer-associated microbiomes [2–4, 36]. However, the exact mechanism by which these bacteria affect the development and progression of colon cancer has not been fully elucidated. In the SCC group, *Porphynomonas*, *Parvimonas*, *Peptoniphilus*, and *Peptococcaceae* were identified. These species are gram-positive anaerobic cocci mainly located among oral bacteria and influence mucosal gene expression, which might contribute to the development of colon cancers [3, 4, 37]. In addition, *Butyricicoccus*, which produces butyrate, was less abundant in the SCC group. Butyrate is an essential metabolite in the human colon and is the preferred energy source because colon epithelial cells have immunomodulatory and anti-inflammatory properties [31]. Some cross-sectional studies reported that, compared to control individuals, patients with colorectal cancer had a lower abundance of butyrate-producing species and lower fecal levels of butyrate, which is consistent with our current data.

Our data clearly suggest that the tumor microbiota between right- and left-sided colorectal cancer patients shows differential microbial diversity and bacterial taxa at several levels, meaning that the RCC and SCC groups may clearly exhibit different specific microbiome compositions.

Genetically, right-sided tumors are commonly associated with microsatellite instability and are highly immunogenic, presenting with BRAF mutations, whereas left-sided tumors show chromosomal instability with mutations in KRAS, APC, PIK3CA, and p53 [9, 11]. In fact, *Fusobacterium nucleatum* signatures in proximal colorectal cancer tissue are correlated with shorter patient survival and molecular alterations such as hypermutation with microsatellite instability and BRAF mutations [3, 38]. Clinically, patients with right-sided tumors present with a worse prognosis than those with left-sided tumors [4, 39].

Therefore, there is a possibility that the gut microbiome affects the development and progression of colon cancer differently according to tumor location.

Although there are a few studies regarding the differences in the tumor microbiota between right- and left-sided colon cancer, compositional alterations in the microbiota are not restricted to cancerous tissue and differ between distal and proximal cancers. [13]. Another report of analysis of on- and off-tumor microbiota suggests that the right and left colon show distinctive bacterial populations; however, the presence of a colonic tumor leads to a more consistent microbiota between locations [13]. Therefore, when we investigate the gut microbiota of colon cancers using feces, we should consider that the findings might be influenced not only by tumor-associated microbiota but also by the surrounding nontumor microbiota.

Because the number of patients enrolled in this study was small, we could not demonstrate clinical characteristics, such as DM, in postcolectomy patients. However, a previous study reported that right hemicolectomy patients rather than left anterior resection patients had higher serum fasting glucose levels than controls, implying that the proximal colon may play an important role in glucose control [20]. In contrast, a large study demonstrated an increased risk of clinically recorded type 2 diabetes among patients who had undergone total and partial colectomy, with the risk being elevated only among individuals who had the left part of their colon removed [14]. Therefore, the gut microbiota has recently been shown to play an important role in the development of metabolic diseases, including obesity and metabolic syndrome [16, 25].

In our current study, the RCR and SCR groups showed clear alterations in microbiome composition. At the family level, *Gemellaceae*, the members of which modulate immunological reactions, was less abundant in the RCR group than in the other groups. In contrast to the RCR group, in the SCR group, a variety of gut bacteria belonging to the *Firmicutes* phylum were abundant, but the abundance of *Fusobacteriacea*e was low. A previous study reported that the ratio of *Firmicutes* to *Bacteroidetes* was significantly lower in patients with DM [20]. However, our study showed a high *Firmicutes-*to-*Bacteroidetes* ratio in the SCR group. Further studies are needed to determine how gut microbiome composition is related to clinical manifestations in patients with colectomy.

Finally, we performed metabolic function prediction of the microbiota by PICRUSt2 to clarify the functional enzyme spectrum in each group. We revealed the differential expression of several genomes related to glucose metabolism in each group. A previous meta-analysis of metagenomic studies identified the microbiome function of gluconeogenesis and the putrefaction and fermentation pathways as being associated with colorectal cancer [40]. Furthermore, we revealed that cholesterol transport- and metabolism-related enzymes were specifically upregulated in the RCC group and that cobalamin metabolism-related enzymes were downregulated in the SCC group. These results supported previous reports in which cholesterol may inhibit the proliferative capacity of certain human colonic adenocarcinomas [41, 42] and plasma vitamin B12 concentrations are associated with the risk of colorectal cancer [41, 43]. Furthermore, some SCFA-related enzymes were identified in both the RCC and SCC groups. However, these specific characteristics were not observed in the postoperative groups. These results support the idea that pathological microbial dysbiosis is responsible for the gut of patients with colorectal cancer. Future shotgun metagenomic studies of the intestinal mucosa-associated microbiome will be important to further refine the list of colorectal cancer-associated gut microbes.

Several limitations associated with the present study warrant mentioning. First, this was a single-center study, and the sample size was relatively small, which may limit the generalizability of the results. Second, this was a cross-sectional study, and the mean age or background of the subjects was somewhat different, which may affect the gut microbiota among the groups. Third, our study analyzed the gut microbiota in subjects who did not consume controlled diets, which may also influence the results.

In conclusion, we clearly found that the composition of the gut microbiota dramatically differs between right and sigmoid colon cancer patients and between right hemicolectomy and sigmoidectomy patients. Our findings support the hypothesis of tumor location-specific microbiota. We found that high richness and diversity were associated with the RCC and SCR groups and the different gut microbiota compositions in each group. It is difficult to determine whether the gut microbiota could influence the pathophysiological condition of patients or whether the gut condition could alter the microbiota. However, in assessing the gut microbiota in patients with colon cancer, we should consider tumor location.

We hope that the results herein can provide useful information for using gut microbes as biomarkers to assess the location and progression of colon cancer or lead to interventional targets to control the development of this disease.

## Data Availability

The datasets generated and/or analyzed during the current study are available in the DDBJ Sequenced Read Archive, [https://www.ddbj.nig.ac.jp/index-e.html], under the accession number DRA013452.

## Abbreviations

BMI: Body mass index
T-RFLP: Terminal restriction fragment length polymorphism
SCFA: Short-chain fatty acid
